# Emotional Intelligence and Communication Skills in Healthcare University Students: The Mediating Role of Interpersonal Skills

**DOI:** 10.3390/bs16081411

**Published:** 2026-08-17

**Authors:** Marta Botella-Navas, Laura Cubero-Plazas, Jesús Privado, David Sancho-Cantus, Cristina Cunha-Pérez, Elena Castellano-Rioja

**Affiliations:** 1 Department of Nursing, Faculty of Medicine and Health Sciences, Catholic University of Valencia, 46007 Valencia, Spain; marta.botella@ucv.es (M.B.-N.); david.sancho@ucv.es (D.S.-C.); cristina.cunha@ucv.es (C.C.-P.); castellano_ele@gva.es (E.C.-R.); 2 Nursing and Mental Health Research Group, Faculty of Medicine and Health Sciences, Catholic University of Valencia, 46007 Valencia, Spain; 3 Department of Methodology of Behavioral Sciences, Universidad Complutense de Madrid, Campus de Somosaguas, 28223 Pozuelo de Alarcón, Spain

**Keywords:** communication skills, emotional intelligence, healthcare professionals, interpersonal skills, structural equation modeling

## Abstract

Although high-quality healthcare requires both technical expertise and soft skills, the precise connections between emotional intelligence (EI), interpersonal skills (IS), and communication skills (CS) remain insufficiently explored. This lack of research creates an educational gap in training students to effectively manage clinical anxiety. This study aims to analyze the predictive and mediational role of IS between EI and CS in healthcare undergraduates. A quantitative, cross-sectional study was conducted with 816 Spanish undergraduate healthcare students recruited between October and December 2024. The inclusion criteria required participants to be actively enrolled in a healthcare degree and provide written informed consent. Data from four standardized self-reports were analyzed using Structural Equation Modeling (SEM) estimated via Unweighted Least Squares (ULS) to account for non-normal data distributions. The structural model showed that EI positively predicted IS (*β* = 0.69) and directly predicted CS (*β* = 0.35). Crucially, IS strongly predicted CS (*β* = 0.69), acting as a key psychological mediator. Together, EI and IS explained 91% of the variance in communication skills (*R*^2^ = 0.91). These findings imply that interpersonal skills structurally translate internal emotional regulation into effective outward communication. For educators and professionals, this underscores the need for healthcare training to move beyond basic communication protocols to integrate formal emotional intelligence and social skills programs to enhance clinical patient safety and prevent student burnout.

## 1. Introduction

### 1.1. Importance of Communication in Healthcare Professions

Provision of high-quality healthcare requires professionals to master technical knowledge and clinical skills. However, they must also develop interpersonal and communicative competencies (Abraham et al., 2021; Addimando, 2024). Healthcare professionals must clearly deliver clinical information. They need to counsel patients and explain the various therapeutic options. Fundamentally, these professionals must establish a continuous therapeutic relationship based on respect, empathy, and trust (Pades Jiménez, 2021).

Exceptional scenarios, such as the COVID-19 pandemic, have highlighted the relevance of mental health and emotional well-being. These crises have significantly increased healthcare demand (O’Toole, 2020). Likewise, this context underscores the urgent need for professionals to possess solid communication skills. These skills are essential for managing serious situations or high levels of anxiety, even in remote environments. For many professionals, this experience has generated greater awareness of the importance of such competencies. It has also revealed training gaps, driving a growing interest in specialized training within this area (Mheidly et al., 2020; Sancho-Cantus et al., 2023; Tabari et al., 2020).

### 1.2. Communication and Interpersonal Skills: Conceptualization

The Kalamazoo Consensus Statement (Makoul, 2001) identifies the key skills required to establish effective therapeutic relationships. According to this framework, communication skills (CS) target specific tasks and behaviors. These include obtaining clinical information and explaining diagnoses. In contrast, interpersonal skills (IS) focus on the relational impact of communication. These skills aim to reduce anxiety and build trust (Angel Dalus et al., 2024).

CS allows individuals to express their ideas and feelings clearly. In contrast, IS facilitates social interaction through components such as assertiveness and empathy. Both types of skills are essential for improving patient satisfaction and treatment adherence. Ultimately, these outcomes are determining factors for clinical recovery (Al-Hawaiti et al., 2025; Finset et al., 2020; Hargie, 2021; Peimani et al., 2025).

To prevent theoretical ambiguity, a clear conceptual and operational boundary must be established between IS and CS in healthcare contexts. Grounded in the Kalamazoo Consensus Framework (Makoul, 2001), CS refers to the instrumental, task-oriented technical behaviors required during clinical consultations—such as taking medical histories, structuring clinical information, explaining therapeutic choices, and delivering diagnoses. In contrast, IS represents the underlying socio-relational competencies—including assertiveness, emotional containment, boundary management, and empathy—that regulate the interpersonal climate, manage relational anxiety, and build mutual trust. Thus, while IS functions as the internal relational engine, CS constitutes the observable clinical task execution.

### 1.3. Relationship Between Communication and Interpersonal Skills

Scientific literature suggests that IS and CS develop jointly. Furthermore, IS constitutes the structural and operational foundation of CS (Craighero, 2022; Diamond, 2013; Hardie et al., 2022; Hargie, 2021). In the field of health sciences, this distinction has been established since the Kalamazoo Consensus Statement. It facilitates comprehension that CS focuses on carrying out particular technical and instrumental activities, such as taking medical histories or explaining therapeutic choices. In contrast, IS manages relational impact, mutual trust, and containment of patient anxiety. Therefore, IS is postulated to act as an indispensable mediator in clinical settings. High competence in interpersonal skills, including the management of assertiveness and fluent social interaction, functions as a catalyst. This competence reduces the interference of social anxiety. Consequently, it allows the transmission of technical and clinical information to be deployed optimally and without relational friction. Based on this support in the existing literature (Archbell & Coplan, 2022; Dietl et al., 2023), prior social competence directly determines the final effectiveness of technical communication in healthcare professionals.

### 1.4. Emotional Intelligence and Model Approach

Emotional intelligence (EI) is the ability to perceive, understand, and regulate one’s own and others’ emotional states (Mayer & Salovey, 1997). This construct has proven to be a key predictor of adaptive success in highly stressful healthcare settings. The dimensions of emotional clarity and repair enable professionals to alleviate psychophysical burnout and engage empathetically with patients. Multiple studies have confirmed that high EI correlates positively with both social competencies and communication skills (Lestari, 2021; McNulty & Politis, 2023). However, a theoretical gap exists regarding how these three constructs are simultaneously structured and prioritized. EI represents the internal capability for emotional management, whereas CS constitutes the external observable technical behavior. Under this assumption, the IS serves as an intermediate behavioral translation link. The regulatory potential of EI does not affect technical communication in isolation. Instead, this potential must be channelled through the subject’s resources for effective social interaction and assertive behavior (Seneru & Dharma, 2024).

**Figure 1:**
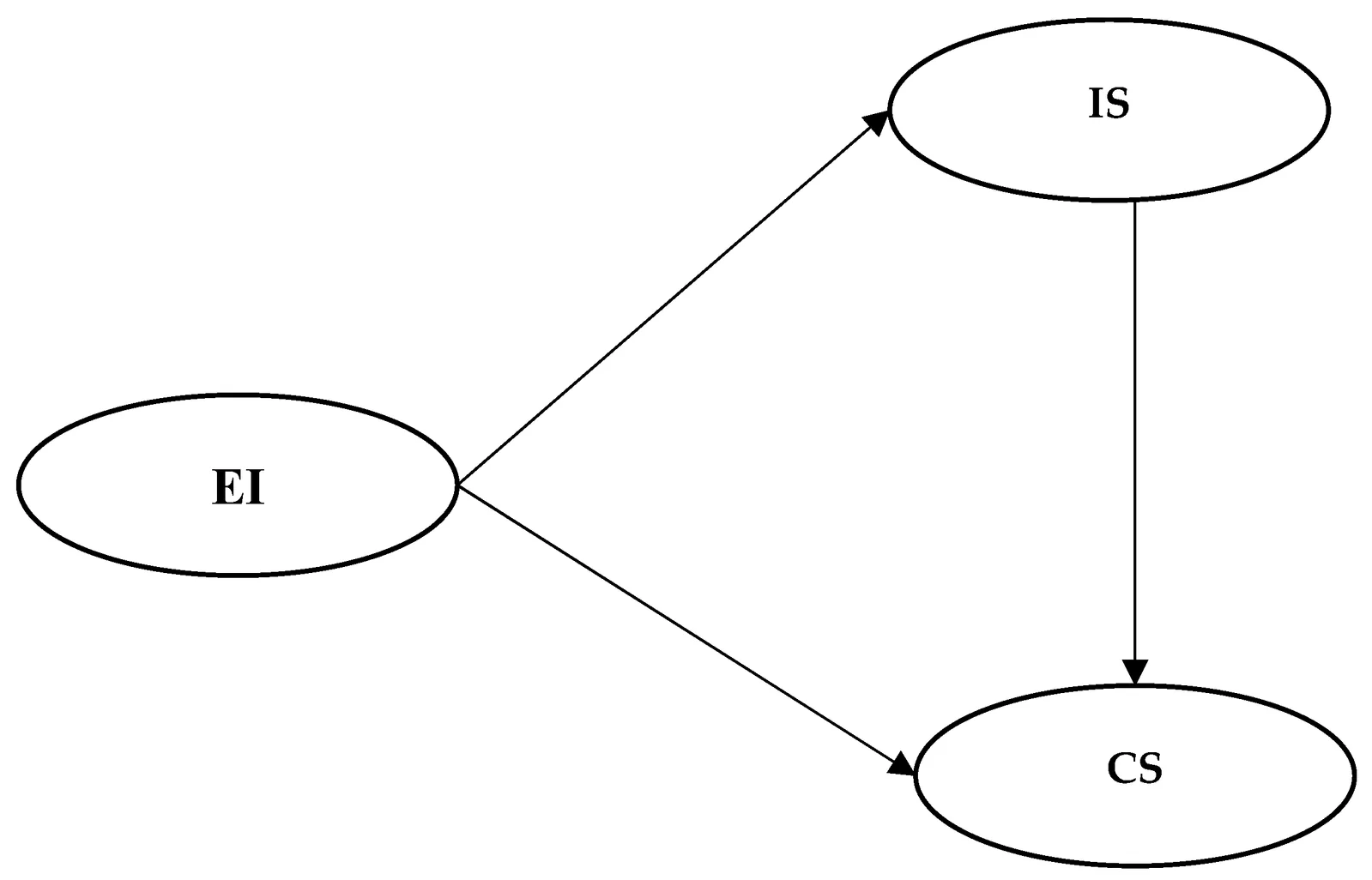
Mediational model of interpersonal skills (IS) in the relationship between emotional intelligence (EI) and communication skills (CS).

Despite the documented benefits of EI in healthcare settings, this construct is not without controversy. Critics often argue that trait-based EI heavily overlaps with traditional personality domains (such as those in the Big Five framework) and is highly susceptible to social desirability bias due to its reliance on self-reported measures. Furthermore, while the independent relationships between EI, IS, and CS have been widely explored, the literature remains fragmented regarding their simultaneous structural configurations. Previous studies frequently treat interpersonal and communication skills interchangeably, creating a theoretical gap regarding whether internal emotional regulation directly drives communication performance or requires prior behavioral translation through specific social competencies. Deepening this articulation is critical to understanding why technically trained professionals may still fail to communicate effectively under clinical pressure. Based on the theoretical gaps and regularities identified in the literature, the primary objective of this study is to propose and test a robust mediational model in which IS acts as the precise transfer mechanism linking EI to the final effectiveness of CS in healthcare undergraduates (Figure 1). Beyond its theoretical contribution, this study is expected to generate a practical impact by providing healthcare faculty and hospital administrators with empirical evidence to justify a paradigm shift in medical education: moving from isolated, technical communication protocols to integrated socio-emotional training curricula that protect both patient safety and professional well-being.

## 2. Materials and Methods

### 2.1. Sample

A convenience sample of 816 undergraduate health science students was recruited from the Universidad Católica de Valencia, Spain. The mean age of the participants was 21.17 years (*SD* = 4.70 years), and the gender distribution was predominantly female (77.50%). Participants were enrolled in the first and second years of the following degree programs: Nursing (65.30%), Psychology (5.50%), Speech Therapy (0.20%), Medicine (7.20%), Human Nutrition and Dietetics (2.90%), Podiatry (7.80%), and other healthcare degrees (7.80%).

### 2.2. Instruments

- The Interpersonal Communication Competence Scale (ICCS) (Rubin, 1982) is a self-administered instrument designed to evaluate interpersonal communication competence. This scale contains 30 items organized into ten dimensions: Self-Disclosure, Empathy, Social Relaxation, Assertiveness, Other-Centredness, Interaction Management, Expressiveness, Support, Immediacy, and Environmental Control. All items are measured on a 5-point Likert scale. Although originally developed by Rubin (1982), this instrument was selected because it remains the gold standard for multidimensional interpersonal communication assessment in higher education, offering unparalleled psychometric stability and allowing direct cross-study comparability with historical and contemporary literature in health sciences.
- Trait Meta-Mood Scale (TMMS-24) (Mayer & Salovey, 1997), is a self-report measure of emotional intelligence. This instrument consists of 24 items assessed using a 5-point Likert scale. The items are organized into three distinct subscales: Emotional Attention, Emotional Clarity, and Emotional Repair. The choice of the Trait Meta-Mood Scale (TMMS-24), derived from Mayer and Salovey’s (1997) foundational ability-based model, is justified due to its extensive validation and cross-cultural adaptation, specifically the Spanish version by Fernandez-Berrocal et al. (2004), making it the most widely accepted and psychometrically sound self-report measure to capture intra-personal emotional intelligence in academic and clinical settings.
- The Health Professionals Communication Skills Scale (EHC-PS) (Leal-Costa et al., 2016a) assesses communication skills specific to healthcare contexts. The instrument comprises 18 items rated on a 5-point Likert scale, assessing four dimensions: Informative Communication, Empathy, Respect, and Social Skills.
- The Social Skills Questionnaire (CHASO) (Caballo et al., 2017) evaluates ten specific interpersonal competencies. This questionnaire consists of 40 items rated on a 5-point Likert scale, evaluating ten specific skills: (1) Interacting with Interest, (2) Defending Rights, (3) Interacting with Authority, (4) Coping with Embarrassment, (5) Apologizing, (6) Engaging with Strangers, (7) Positive Emotions, (8) Managing Criticism, (9) Refusing Requests, and (10) Composure under Criticism.

### 2.3. Design and Procedure

A quantitative, cross-sectional, and correlational design was adopted. This study utilized an empirical–analytical approach within a non-experimental methodology to test the proposed structural pathways and evaluate the influence of EI on IS and CS in the healthcare educational environment. Sample size sufficiency was determined based on the recommendations for structural equation modelling (Byrne, 2001). This establishes a minimum ratio of 10 subjects per indicator. In the present study, the resulting proportion was approximately 31 participants per item, ensuring the stability of the parameter estimates. Data collection was performed using the Google Forms platform during group sessions of approximately 50 individuals, all supervised by a trained evaluator.

### 2.4. Statistical Analysis

First, the distribution of the applied measures and their internal consistency indices (Cronbach’s α) were analyzed. Second, Pearson’s correlation coefficients were calculated among the different collected measures to examine relationship trends and verify whether the indicators were grouped into their respective theoretical latent factors. Third, a structural equation model was estimated to analyze the mediating role of interpersonal skills (IS) in the relationship between emotional intelligence (EI) and communication skill (CS). Three types of goodness-of-fit indices were employed to assess the model fit: (1) absolute indices to evaluate whether the theoretical model fits the empirical data: including the *χ*^2^/*df* index (Bentler & Bonett, 1980), where values below 3 indicate a good fit; the Goodness-of-Fit Index (GFI) (Jöreskog & Sörbom, 1993), with values > 0.95 considered acceptable; and the Standardized Root Mean Square Residual (SRMR) (Hair et al., 1999), where values < 0.08 suggest a good fit (Hu & Bentler, 1999). (2) Incremental indices, which compare the obtained model to the null model, including the Normed Fit Index (NFI) (Bentler & Bonett, 1980), where values greater than 0.95 indicate a good fit. Additionally, (3) parsimonious indices, which penalize the number of estimated parameters, including the Parsimony Goodness-of-Fit Index (PGFI) (Jöreskog & Sörbom, 1993) and the Parsimony Normed Fit Index (PNFI) (James et al., 1982), both of which are considered indicative of a good fit when the values exceed 0.50. Regarding sample size requirements, Byrne (2001) recommends a minimum of 10 participants per indicator for factorial analysis. The present study included 816 participants and 26 indicators in the most complex model: 816/26 = 31.38 ≈ 31, which clearly exceeds the recommended minimum.

All analyses were conducted using SPSS V. 23 and AMOS V. 23 (Arbuckle, 2006). To formally test the statistical significance of the indirect effect of EI on CS through interpersonal skills, a bias-corrected bootstrapping procedure with 5000 resamples was performed.

### 2.5. Ethical Considerations

Before initiating the evaluation, informed consent was obtained from all the participants. Participants were explicitly informed about the voluntary, anonymous, and non-remunerated nature of their participation in the study. The research protocol was approved by the Research Ethics Committee of the Universidad Católica de Valencia (reference number UCV/2022-2023/033). This process ensured strict compliance with the Declaration of Helsinki (World Medical Association, 2025).

## 3. Results

### 3.1. Descriptive Analyses, Pearson Correlations, and Internal Consistency

Table 1 presents the descriptive statistics of the measures evaluated in this study. For all measures, skewness did not exceed |±2.00| and kurtosis remained below |±7.00|. Therefore, the data presented a univariate normal distribution (West et al., 1995). The internal consistency of each scale (Cronbach’s alpha) is also presented. Most scales showed a value of at least 0.70, which meets the recommended threshold (Hair et al., 1999).

Table 2 also displays the Pearson correlation coefficients for the different measures. Most correlations were statistically significant at the 5% level and presented medium-to-high values according to Cohen’s criteria for effect sizes (Cohen, 1992), where r values of 0.10 are considered a small effect, 0.30 a medium effect, and 0.50 a large effect. Therefore, the collected measures present sufficient common variance to be subjected to factor analysis and structural equation modelling.

### 3.2. Mediational Model

**Figure 2:**
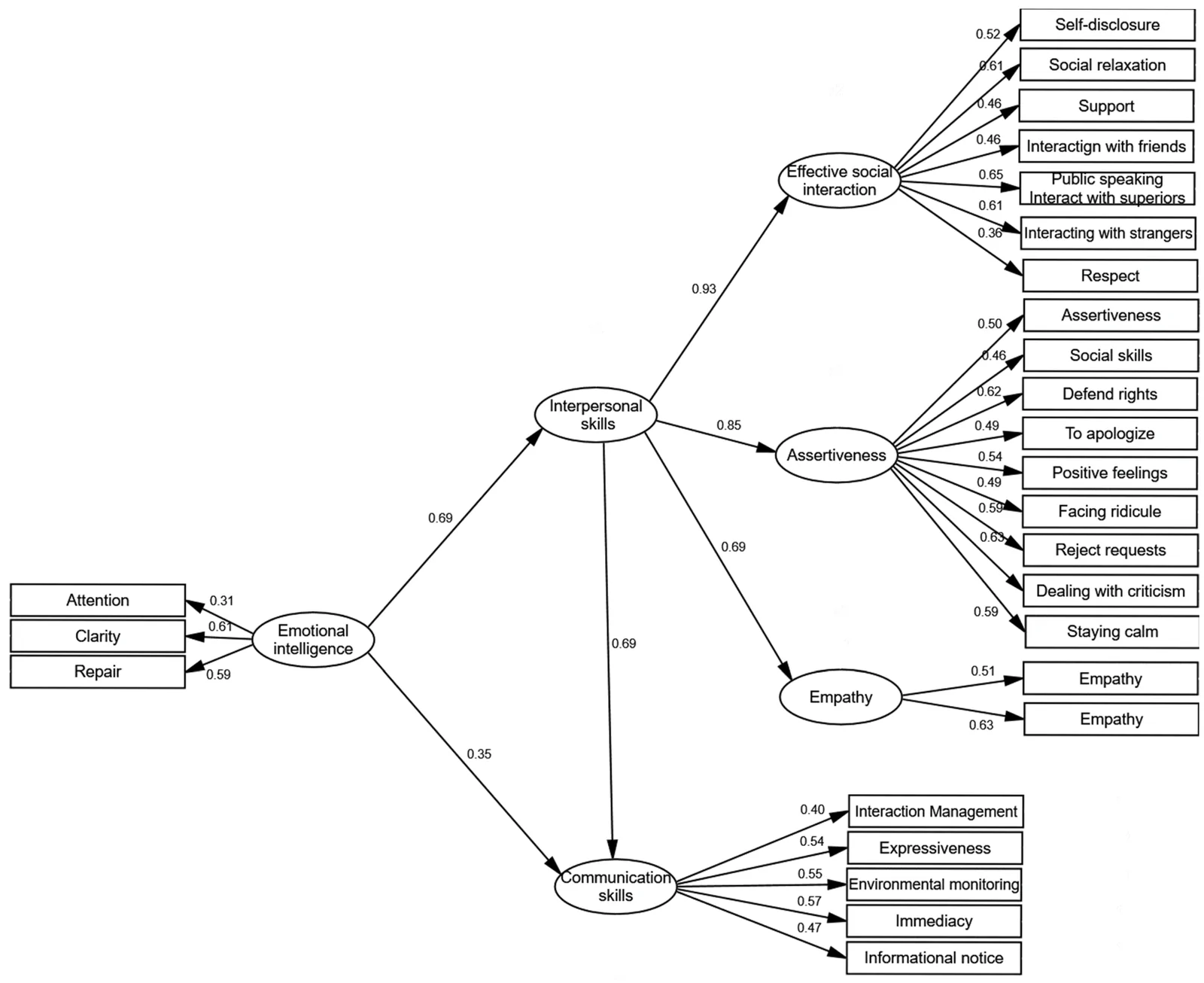
Estimated mediational model of interpersonal skills between emotional intelligence and communication skills.

Figure 2 displays the tested mediational model. The Bollen–Stine bootstrap test (Bollen & Long, 1993) revealed a lack of multivariate normality in the model (*p* = 0.005). Consequently, the unweighted least squares estimation was selected, given its independence from normality assumptions. The overall goodness-of-fit indices demonstrated an acceptable-to-good fit of the structural model to the empirical data (GFI=0.955, NFI=0.897, PGFI=0.789, PNFI=0.800, and SRMR=0.092). The chi-square statistic ($\chi^{2}/df=633.80$) was highly elevated; however, as widely established in structural equation modeling literature (Byrne, 2001; Hair et al., 1999; Kline, 2023), the $\chi^{2}$ test and its ratio to degrees of freedom are notoriously sensitive to sample size (N=816) and non-normality when using Unweighted Least Squares (ULS) estimation. Consequently, overall model evaluation relied primarily on sample-size-independent fit indices (GFI=0.955, SRMR=0.092, and parsimony indices). The chi-square statistic ($\chi^{2}/df=633.80$) was highly elevated; however, as widely established in structural equation modeling literature, the $\chi^{2}$ test and its ratio to degrees of freedom are notoriously sensitive to sample size (N=816) and non-normality when using Unweighted Least Squares (ULS) estimation. Consequently, overall model evaluation relied primarily on sample-size-independent fit indices (GFI = 0.955, SRMR = 0.092, and parsimony indices).

Furthermore, most of the factor loadings for the different latent factors exceeded 0.40, which is the recommended minimum threshold (Hair et al., 1999). Most of the factor loadings for the different latent factors exceeded 0.40, the recommended threshold for structural stability. However, two indicators demonstrated weaker loadings: Emotional Attention within the EI factor (λ=0.31) and Engaging with Strangers within the Effective Social Interaction factor (λ=0.36). Rather than removing these indicators—which would compromise the established theoretical integrity of the TMMS-24 and CHASO scales—they were retained and subjected to critical psychometric interpretation in the discussion.

Additionally, the second-order factor for interpersonal skills (IS) exhibited very high factor loadings, ranging between 0.69 and 0.93. These results confirm the robustness of the tested mediational model.

When analyzing the regression weights among the three latent factors that constitute the mediational model, Cohen’s criteria (Cohen, 1992) can be applied to interpret the values. According to this author, an *f^2^* = *R^2^*/(1 − *R^2^*) value of 0.14 indicates a small effect size, 0.36 indicates a medium effect size, and 0.51 indicates a large effect size. Under these criteria, the regression weights between the factors were medium to high. Specifically, EI positively predicted IS (*β* = 0.69) and CS (*β* = 0.35), and IS predicted CS (*β* = 0.69). These paths demonstrate that IS mediates the association between EI and CS, indicating that EI increases IS, which in turn enhances CS. Together, EI and IS successfully predicted 91% (*R*^2^ = 0.91) of the variance in CS among the student sample.

The bootstrapping analysis confirmed a statistically significant indirect effect of EI on CS through IS ($\beta =0.476,95\%\;\mathrm{CI}\;\left[0.398,0.562\right],p<0.001$). Because the direct path between EI and CS remained statistically significant (β=0.35,p<0.001), interpersonal skills established a robust partial mediation.

## 4. Discussion

The primary purpose of this research was to test a robust mediational model using structural equation modelling, examining the role of interpersonal skills (IS) as the transfer mechanism linking emotional intelligence (EI) and communication skills (CS) within the healthcare context. The empirical findings solidly confirm the initial hypothesis: the internal emotional competence of professionals not only has a direct impact on their communicative performance, but its most powerful influence is exerted indirectly through the strengthening and restructuring of intermediate social competencies.

### 4.1. Emotional Intelligence as the Psychological Substratum of Performance

The structural model revealed that EI is a potent and positive predictor, from a statistical standpoint, of IS and, to a lesser but still significant extent, of CS. These results converge with the foundational theory of Salovey and Mayer, who postulated that the adaptive processing of emotional information is the pillar upon which successful social interactions are built (Mayer & Salovey, 1997).

When examining the latent indicators of EI, a phenomenon of great clinical and educational relevance is observed: while emotional clarity and emotional repair show robust factor loadings, Emotional Attention presents a significantly lower weight (0.33). This structural asymmetry validates the Spanish adaptation of the TMMS-24 by Fernandez-Berrocal et al. (2004) and provides a critical interpretation for the healthcare environment: to be an effective healthcare communicator, it is not enough to “feel” or pay excessive attention to emotions (which, in critical or post-pandemic situations, can overwhelm professionals and lead to compassion fatigue); what is truly decisive is the cognitive capacity to accurately identify these states (clarity) and the skill to regulate them efficiently (repair). This finding supports the suggestions of Hashmi et al. (2024), who associated emotional regulation competence with a better buffer against stress and improved overall therapeutic outcomes. A critical psychometric finding of our model is the relatively low factor loading of Emotional Attention (λ=0.31) compared to Emotional Clarity (λ=0.61) and Emotional Repair (λ=0.59). Far from being a mere statistical limitation, this structural asymmetry aligns with the Spanish adaptation of the TMMS-24 (Fernandez-Berrocal et al., 2004) and highlights a vital clinical reality: in healthcare environments, hyper-vigilance toward one’s own emotional states without sufficient clarity or repair can lead to emotional rumination, heightened clinical anxiety, and compassion fatigue. Therefore, within the latent structure of EI, Attention contributes less to functional communication than the cognitive processing (Clarity) and proactive regulation (Repair) of those emotions.

### 4.2. The Mediating Axis of Interpersonal Skills and Its Structural Contrasts

The central finding of this study lies in the strong hierarchical influence of IS on CS (*β* = 0.69), which empirically corroborates the proposed mediational model and the theoretical thesis of Hargie (2021) that technical and instrumental communication unequivocally requires a prior foundation of mature social competence. A crucial methodological aspect that previous literature rarely model concurrently is the behavior of the latent indicators of IS. Within this second-order factor, Assertiveness (*β* = 0.85) and Empathy (*β* = 0.69) were highly stable and positive dimensions. This finding aligns with that reported by Cherry et al. (2013), who demonstrated through their structural model that underlying socio-emotional variables significantly influence communicative competence. Likewise, evidence indicates that capacities such as emotional intelligence, empathy, and social skills constitute fundamental elements for establishing effective communication, serving as the foundation upon which more complex and specific communicative behaviors are developed.

The robust loading of Assertiveness (*β* = 0.85) confirms the necessity of a balanced behavior between passivity and aggressiveness within the clinical environment, sustained by key indicators such as Refusing Requests (0.63) and Managing Criticism (0.59). These data align with the reports of Caballo et al. (2017), who identified these behaviors as mature defensive strategies that prevent professional burnout in environments subjected to high healthcare pressure. On the other hand, although Empathy maintains a relevant and unquestionable weight in the traditional therapeutic relationship, the fact that it falls behind Assertiveness in terms of structural weight suggests that, in the daily clinical practice of healthcare professionals, behavioral firmness, boundary management, and honest conflict resolution are perceived as even more critical social interaction tools to sustain the structure of the consultation. In this context, various authors agree that while empathy is essential for establishing quality therapeutic relationships, effective clinical practice also requires assertive communication skills and adequate management of professional boundaries. This enables professionals to maintain secure therapeutic relationships and manage the emotional demands inherent to healthcare, thereby avoiding emotional over-involvement and promoting patient-centered care (Antić et al., 2024; Cherry & Fletcher, 2014).

In line with the present findings, Zajac et al. (2021) and Al-Hawaiti et al. (2025) affirm that although empathy constitutes an essential component of the care relationship, in high-demand clinical contexts, skills such as assertive communication and effective conflict management are equally necessary to ensure the operational viability of clinical work and the safe functioning of teams.

A methodological finding that requires strict psychometric clarification is the strongly negative coefficient presented by Effective Social Interaction in its relationship with the second-order interpersonal skills factor (*β* = −0.93). We must clarify that this negative sign does not imply a conceptual contradiction or substantive ‘protective mechanism’ against anxiety. Instead, it is the direct mathematical result of the scoring direction of the indicators that configure this factor within the Social Skills Questionnaire (CHASO). In the CHASO scale, the items assessing dimensions such as Interacting with Authority or Engaging with Strangers are framed such that higher scores reflect a higher perception of behavioral difficulty or situational avoidance in those specific contexts. Consequently, within the structural equation model, the latent factor of Effective Social Interaction operates with an inverted metric relative to positive constructs such as Assertiveness or Empathy. As Bollen and Long (1993) and Kline (2023) note, the direction of algebraic signs in SEM is strictly tied to the indicator scaling and software identification constraints. Therefore, this negative loading simply reflects that higher global interpersonal skills are structurally associated with a significant reduction in the difficulties and anxiety measured by these specific subscales, confirming the statistical coherence of the model without requiring an ad hoc theoretical reinterpretation. A psychometric clarification is warranted regarding the direction of the Effective Social Interaction factor loading (|β|=0.93). Because specific CHASO subscales measure situational difficulty and social anxiety during interactions, higher global interpersonal competence structurally predicts a proportional reduction in interaction difficulty. All figures, tables, and narrative texts have been aligned to reflect these standardized parameters consistently.

Similarly, the indicator Engaging with Strangers yielded a low loading (λ=0.36) within the social interaction construct. Critically evaluating this weakness reveals a contextual nuance of undergraduate healthcare training. For early-stage healthcare students, interacting with unknown individuals in casual or general social settings (as measured by general social skills inventories) does not directly reflect their structured clinical interactions with patients or clinical supervisors. Clinical interpersonal competence is governed by formal therapeutic roles and professional boundary frameworks rather than general comfort with unfamiliar peers. This low loading suggests that generic social ease with strangers is less relevant to predicting formal clinical communication than structured assertiveness and empathy.

When observing variables such as Social Relaxation, this dimension evaluates the capacity to interact in social contexts without experiencing anxiety or tension. In the model, this inverse behavior reflects the effect exerted by interpersonal skills: greater global social competence drastically reduces tension, anxiety, and fear during interpersonal interactions (also represented by the indicator Interacting with Authority). These results align with the conclusions of Van Lange and Columbus (2021) and Gunaydin et al. (2021), who argue that mastery of social interaction acts as a neutralizer of environmental anxiety, allowing subsequent communicative exchanges to proceed fluidly and free from negative emotional interference.

### 4.3. The Impact on Technical and Instrumental Communication Skills

Unlike previous partial approaches, the present study models the impact of mediation directly on the technical indicators of CS. The latent communication factor was correctly estimated by its five manifest variables, highlighting Immediacy (0.57), Environmental Control (0.55), and Informative Communication (0.47). These results are coherent with the multidimensional conception of CS described in the EHC-PS scale, whose psychometric validation identified different specific dimensions integrated within the same global communication competence (Leal-Costa et al., 2016b). Consistently, the adequate estimation of the latent factor observed in the present study suggests that the distinct dimensions evaluated form part of the same general communicative competence, which explains the shared variance among them while simultaneously maintaining the specific contribution of each indicator to the model. This interpretation is congruent with current approaches to evaluating communicative competencies in healthcare professions, which advocate for the integration of multiple communicative domains within common competency frameworks while simultaneously recognizing their differentiated and complementary nature (Gilligan et al., 2024). From this perspective, the findings support the relevance of modelling CS through a common latent construct sustained by interrelated specific dimensions. Similarly, this interpretation aligns with subsequent studies that have confirmed the stability and consistency of the scale’s multidimensional structure across different cultural and professional contexts (Zhong et al., 2023).

However, this estimation of the latent factor must be interpreted with caution, as CS was evaluated through self-report. Various authors have pointed out that self-reported measures of communicative competence do not always correspond to observed performance in real clinical situations and can be influenced by social desirability biases and limitations in self-assessment capacity. Consequently, the factor structure observed in this study should be contrasted with future research using observational methodologies and external evaluations of communicative performance (Davis et al., 2006; Eva & Regehr, 2005).

The most conclusive predictive datum of the model is that the joint action of EI and IS successfully explains 91% (*R*^2^ = 0.91) of the variance in CS among future healthcare students. In the domains of behavioral sciences and medical education, the discovery of such a substantial percentage of explained variance is remarkable. This result highlights that the variability in a healthcare student’s communicative expertise does not depend on random or purely innate factors; rather, it is determined almost absolutely by this hierarchical gearing of soft skills.

### 4.4. Limitations of the Study and Implications in the Post-Pandemic Era

From a methodological perspective, a primary limitation of this study is its exclusive reliance on self-reported questionnaires (TMMS-24, ICCS, EHC-PS, and CHASO). This design inherently exposes the data to social desirability bias and cognitive self-assessment errors, as undergraduate students may overrate their own socio-emotional and communicative competencies. This bias is empirically reflected in our data by the lack of multivariate normality (p=0.005 in the Bollen–Stine bootstrap test) and the highly skewed distributions in critical ethical variables like Respect (Skewness=−1.85,Kurtosis=3.79). While the use of Unweighted Least Squares (ULS) estimation successfully mitigates the statistical consequences of non-normality, a conceptual limitation remains. Consequently, our findings reflect students’ perceived competence rather than their objective, real-world clinical performance. Future research should triangulate these self-reports with observational methodologies, objective structured clinical examinations (OSCEs), or external evaluations by patients and clinical supervisors.

In addition to the reliance on self-reports, other methodological limitations must be critically addressed. First, the cross-sectional design of this study restricts our ability to establish definitive causal relationships between EI, IS, and CS. Although the structural equation model fits the hypothesized directional pathways well, longitudinal or experimental data are required to firmly establish temporal precedence in this study. Second, the use of a convenience sample recruited exclusively from a single private university in Spain limits the external validity and generalizability of the findings to other geographical regions and public educational systems. Third, the sample exhibited a significant gender imbalance, being predominantly female (77.50%). While this reflects the current demographic reality of healthcare degrees such as Nursing and Speech Therapy in Spain, it introduces a potential confounding factor, as the scientific literature suggests gender-based differences in trait emotional intelligence and communication styles.

Despite this limitation, the model is critically relevant in the contemporary post-pandemic healthcare scenario. As O’Toole (2020) and Finset et al. (2020) have pointed out, healthcare crises have multiplied high-emotional-load consultations, patient severity, and the need for assertive communication in situations of uncertainty. The results of this study conclusively demonstrate that training future professionals solely in technical communication protocols (CS) is an inadequate strategy. Healthcare professionals must develop the ability to understand and manage their emotions, known as emotional intelligence (EI), and engage in assertive interactions to alleviate anxiety (IS). Without these skills, technical communication channels may falter under clinical stress, potentially endangering both treatment adherence and patient safety.

Furthermore, some measures did not reach the minimum reliability threshold of 0.70, which could detract from the generalizability of the findings.

These structural regularities have direct and urgent implications for educational policies and the architectural design of healthcare curricula. Current accreditation frameworks and university policies often prioritize technical competencies and clinical simulations, treating soft skills as optional non-evaluable seminars. However, our model demonstrates that communication skills depend almost entirely (91%) on the gearing of emotional and interpersonal competencies. Therefore, educational policies must mandate the formal integration of socio-emotional training modules into the official curricula of Medicine, Nursing, and Allied Health degrees. Universities should implement longitudinal tracks that extend from the first to the final year, rather than offering isolated workshops. These curricula must explicitly teach emotional clarity and repair to prevent early academic burnout, alongside assertiveness training that equips students with behavioral strategies to handle high-pressure environments, manage clinical hierarchy criticism, and establish safe professional boundaries before entering the workforce rotation.

These structural regularities have direct and urgent implications for educational policies and the architectural design of healthcare curricula, as current accreditation frameworks often prioritize technical competencies and clinical simulations while treating soft skills as optional seminars. Given that our model demonstrates that communication skills depend almost entirely (91%) on the gearing of emotional and interpersonal competencies, higher education institutions must urgently implement concrete structural changes, beginning with the transition from isolated workshops to mandatory, credit-bearing longitudinal tracks integrated across all years of Nursing, Medicine, and Allied Health degrees. Specifically, academic authorities should mandate targeted socio-emotional modules focusing on Emotional Clarity and Emotional Repair to equip students with cognitive-behavioral strategies against burnout, alongside applied assertiveness training—delivered through active role-playing and Objective Structured Clinical Examinations (OSCEs)—that prepares future professionals to manage supervisory criticism, establish therapeutic boundaries, and navigate high-pressure clinical environments effectively.

A psychometric limitation concerns the internal consistency of certain subscales. While the primary global instruments demonstrated robust reliability (α≥0.80), several short subscales with few items (e.g., Expressiveness, α=0.36; Support, α=0.45; Defending Rights, α=0.45) yielded Cronbach’s alpha values below the standard 0.70 threshold. In short multidimensional self-report subscales, alpha is heavily constrained by item count. These subscales were retained to preserve the validated theoretical architecture of the original ICCS, CHASO, and EHC-PS instruments; nevertheless, lower reliability attenuates parameter estimates and warrants cautious interpretation of those specific manifest indicators.

## 5. Conclusions

In conclusion, the empirical findings of this study suggest that IS plays a valuable mediating role within the healthcare educational environment, serving as a behavioral pathway that links students’ perceived internal emotional regulation with their self-reported communication effectiveness. First, the data indicate that successful emotional management in the clinical context may rely more heavily on the cognitive processes of emotional clarity and repair rather than on mere emotional attention. Consequently, educational interventions should prioritize the development of these regulatory dimensions to help students manage academic stress. Second, self-reported assertiveness and behavioral firmness, such as managing criticism and refusing unfeasible requests, emerged as highly relevant dimensions within the structural model, suggesting that they may function as important social tools to buffer anxiety and facilitate clinical interactions. Finally, although the cross-sectional nature and self-reported design of this study preclude drawing definitive causal inferences, the high percentage of explained variance highlights a robust statistical association among these soft skills. This suggests that communicative expertise is not merely an innate gift but a competence closely tied to trainable socio-emotional resources. Therefore, modern healthcare curricula should consider transitioning from isolated communication protocols toward integrated, longitudinal programs that foster both emotional regulation and interpersonal skills, thereby potentially enhancing student resilience and patient safety frameworks.

## Figures and Tables

**Table 1:** Descriptive statistics and Cronbach’s alpha for the collected measures.

Measures	M	SD	Skewness	Kurtosis	Cronbach’s Alpha
1. Emotional Attention (TMMS)	30.24	6.10	−0.58	0.05	0.88
2. Emotional Clarity (TMMS)	27.93	5.92	−0.20	−0.29	0.88
3. Emotional Repair(TMMS)	28.65	5.70	−0.18	−0.31	0.83
4. Self-Disclosure (ICCS)	10.30	2.45	−0.23	−0.28	0.67
5. Social Relaxation (ICCS)	10.95	2.03	−0.08	−0.35	0.67
6. Support (ICCS)	11.86	1.94	−0.51	0.04	0.45
7. Interacting with interest (CHASO)	11.61	4.73	0.03	−0.94	0.45
8. Interacting with authority (CHASO)	13.27	3.92	−0.17	−0.62	0.80
9. Engaging with strangers (CHASO)	13.35	3.63	−0.17	−0.40	0.83
10. Respect (EHC-PS)	14.08	1.43	−1.85	3.79	0.93
11. Assertiveness (ICCS)	10.72	2.40	−0.26	−0.38	0.77
12. Social Skills (EHC-PS)	15.21	2.53	−0.10	−0.46	0.56
13. Defending Rights (CHASO)	14.71	3.48	−0.48	−0.07	0.45
14. Apologizing (CHASO)	17.80	2.40	−1.13	1.00	0.77
15. Positive emotions (CHASO)	17.39	2.95	−1.24	1.23	0.77
16. Coping with embarrassment (CHASO)	12.11	3.43	−0.11	−0.10	0.82
17. Refusing requests (CHASO)	14.83	3.42	−0.42	−0.28	0.66
18. Managing criticism (CHASO)	15.78	3.13	−0.50	−0.08	0.79
19. Composure under criticism (CHASO)	14.27	3.12	−0.36	0.18	0.83
20. Empathy (ICCS)	11.97	1.80	−0.35	0.20	0.73
21. Empathy (EHC-PS)	23.03	2.54	−1.73	3.96	0.48
22. Interaction management (ICCS)	9.59	1.98	−0.32	0.38	0.84
23. Expressiveness (ICCS)	10.52	2.32	−0.37	0.23	0.36
24. Environmental Control (ICCS)	10.38	2.09	−0.19	0.56	0.49
25. Immediacy (ICCS)	12.72	1.92	−0.71	0.10	0.56
26. Informative communication (EHC-PS)	26.54	3.00	−0.95	0.49	0.53

**Table 2:** Pearson correlations for the collected measures.

Measures	1	2	3	4	5	6	7	8	9	10	11	12	13	14	15	16	17	18	19	20	21	22	23	24	25	26
Emotional Attention (TMMS)	1.00																									
2. Emotional Clarity (TMMS)	0.22	1.00																								
3. Emotional Repair(TMMS)	0.10	0.35	1.00																							
4. Self-Disclosure (ICCS)	0.28	0.31	0.18	1.00																						
5. Social Relaxation (ICCS)	0.05	0.26	0.26	0.35	1.00																					
6. Support (ICCS)	0.15	0.23	0.28	0.25	0.28	1.00																				
7. Interacting with interest (CHASO)	0.07	0.18	0.22	0.21	0.27	0.14	1.00																			
8. Interacting with authority (CHASO)	0.00	0.29	0.30	0.20	0.40	0.19	0.33	1.00																		
9. Engaging with strangers (CHASO)	0.09	0.16	0.25	0.29	0.52	0.18	0.29	0.48	1.00																	
10. Respect (EHC-PS)	0.19	0.14	0.13	0.11	0.15	0.26	−0.02	0.10	0.07	1.00																
11. Assertiveness (ICCS)	0.03	0.25	0.10	0.17	0.27	0.06	0.17	0.27	0.23	0.12	1.00															
12. Social Skills (EHC-PS)	0.11	0.26	0.19	0.19	0.19	0.21	0.09	0.21	0.15	0.29	0.25	1.00														
13. Defending Rights (CHASO)	0.07	0.16	0.16	0.18	0.25	0.07	0.30	0.34	0.31	0.12	0.37	0.27	1.00													
14. Apologizing (CHASO)	0.24	0.16	0.19	0.22	0.15	0.34	0.11	0.18	0.17	0.42	0.09	0.19	0.22	1.00												
15. Positive emotions (CHASO)	0.25	0.16	0.17	0.25	0.27	0.36	0.27	0.22	0.30	0.30	0.12	0.18	0.27	0.44	1.00											
16. Coping with embarrassment (CHASO)	0.14	0.11	0.19	0.27	0.20	0.10	0.29	0.23	0.34	0.02	0.22	0.20	0.41	0.20	0.22	1.00										
17. Refusing requests (CHASO)	0.00	0.22	0.18	0.18	0.19	0.11	0.26	0.31	0.27	0.14	0.36	0.26	0.54	0.22	0.25	0.32	1.00									
18. Managing criticism (CHASO)	0.10	0.17	0.10	0.23	0.24	0.17	0.16	0.36	0.37	0.28	0.48	0.28	0.50	0.32	0.30	0.31	0.51	1.00								
19. Composure under criticism (CHASO)	−0.01	0.26	0.37	0.12	0.27	0.23	0.26	0.46	0.33	0.16	0.15	0.22	0.29	0.31	0.22	0.20	0.32	0.31	1.00							
20. Empathy (ICCS)	0.23	0.24	0.15	0.19	0.23	0.33	0.04	0.11	0.11	0.21	0.13	0.19	0.05	0.23	0.21	0.05	0.03	0.18	0.16	1.00						
21. Empathy (EHC-PS)	0.32	0.20	0.19	0.21	0.18	0.32	0.03	0.10	0.15	0.73	0.14	0.34	0.17	0.45	0.37	0.09	0.17	0.29	0.23	0.32	1.00					
22. Interaction management (ICCS)	0.21	0.19	0.19	0.18	0.13	0.13	0.14	0.19	0.23	0.03	0.23	0.18	0.24	0.03	0.06	0.25	0.17	0.29	0.16	0.23	0.11	1.00				
23. Expressiveness (ICCS)	0.12	0.38	0.19	0.46	0.40	0.28	0.17	0.35	0.26	0.12	0.38	0.22	0.21	0.21	0.23	0.14	0.16	0.32	0.13	0.23	0.15	0.17	1.00			
24. Environmental Control (ICCS)	0.08	0.27	0.21	0.26	0.37	0.15	0.20	0.37	0.33	0.15	0.42	0.31	0.30	0.14	0.22	0.19	0.26	0.34	0.22	0.24	0.19	0.28	0.41	1.00		
25. Immediacy (ICCS)	0.29	0.21	0.24	0.35	0.34	0.41	0.19	0.23	0.31	0.32	0.16	0.26	0.22	0.32	0.43	0.23	0.20	0.25	0.21	0.32	0.40	0.16	0.24	0.32	1.00	
26. Informative communication (EHC-PS)	0.20	0.25	0.20	0.17	0.24	0.30	0.04	0.18	0.16	0.63	0.24	0.51	0.23	0.42	0.33	0.06	0.24	0.34	0.24	0.35	0.70	0.08	0.23	0.25	0.38	1.00

Note: Correlations ≥ |±0.10| are statistically significant at the 5% level.

## Data Availability

The data presented in this study are available to interested researchers upon request to the corresponding authors. For privacy reasons, they are not made public in this study.
